# Pediatric outpatient non-invasive ventilation program: Reflections on the first experience from the Middle East

**DOI:** 10.5339/qmj.2023.5

**Published:** 2022-12-30

**Authors:** Amal AlNaimi, Sara G. Hamad, Rania Arar, Ricardo Mandanas, Mutasim Abu-Hassan

**Affiliations:** ^1^Sidra Medicine, Doha-Qatar. E-mail: shamad@sidra.org ORCID: E-mail: https://orcid.org/0000-0002-8292-2011; ^2^Hamad General Hospital, Doha-Qatar.

**Keywords:** Non-invasive ventilation, outpatient, pediatric

## Abstract

Long-term noninvasive ventilation (NIV) is being progressively used in children. The current guidelines recommend NIV initiation in the hospital during overnight polysomnography (PSG) titration study. Due to the logistic, socioeconomic, and financial difficulties including bed availability, an outpatient program for NIV initiation and patients/parents counseling to prevent delay and provide cost-effective care has been commissioned. Hence, this study reports on the clinical outcome of our program as it represents the first reported experience from the middle east.

A retrospective review of electronic medical charts was conducted for all patients with PSG-confirmed sleep-related breathing disorders (SRBD) who were evaluated and treated in the NIV clinic in the pediatric pulmonary clinic at Sidra Medicine from January 2020 to November 2021. Patients’ data included demographics, PSG results, and NIV clinic records. The results show that twenty-eight patients (17 male, 11 female) were included during the study period. The patients’ median age at NIV initiation was 11 ± 5.17 years. The median BMI was 32.72 ± 15.91 kg/m^2^. The most common diagnosis was morbid obesity in 9 (32%) of the patients. The identified SRBD based on the diagnostic PSG were obstructive sleep apnea in 21 patients, hypoventilation in 3 patients, mixed apnea in 3 patients, and central apnea in 1 patient. The median total Apnea-Hypopnea Index (AHI) was 12.7 (0.7-153.9) events per hour. The main reason for the initial NIV clinic visit was NIV initiation in 19 patients. Upon follow-up, six patients were successfully weaned off NIV support by the treating pulmonologist. Five patients refused to use NIV at home. Fifteen out of the remaining seventeen patients used NIV ≥  4 hours per night (subjective adherence: 88%). Twelve patients used the NIV for ≥  24 nights per month (70.6%). All parents reported that the clinic experience was beneficial and helped them to be more comfortable with applying the NIV machine at home. In conclusion, pediatric NIV outpatient programs are achievable and beneficial, especially in developing countries. Pediatric NIV clinics managed by a specialized and experienced team play important role in the initiation and follow-up of NIV support. They provide alternative pathways for the care of pediatric patients, education, and support of their families, and decrease the load on intensive care and sleep laboratory units. Studies about cost-effectiveness are needed to evaluate the impact of implementing outpatient NIV programs on sleep laboratories and hospital admission rates.

## Introduction

Long-term noninvasive ventilation (NIV) is being progressively used in children with chronic respiratory failure, especially in patients with chest wall disorders and neuromuscular diseases. NIV is also indicated in children with severe upper airway obstruction, particularly in children with complex craniofacial malformations or persistent obstructive sleep apnea (OSA). It plays an essential role in improving the quality of life and increasing the life expectancy in these patients.^1^

The current guidelines of the American Academy of Sleep Medicine (AASM) recommend NIV initiation in the hospital during overnight polysomnography (PSG) titration study.^2,3^ AASM also recommends that all patients should receive adequate NIV Education, hands-on demonstration, mask fitting, and acclimatization before titration. AASM provides a titration algorithm for adult and pediatric patients, which should be implemented by a registered polysomnographic technologist and reviewed by a board-certified sleep specialist.^4^

In children, NIV initiation in the hospital represents the standard practice. However, this is not easily feasible due to the shortage of hospital beds; especially as encountered during the global COVID-19 pandemic, limited access to PSG, and patient/family difficulties.^5^ Moreover, the increased frequency of hospitalizations may be stressful for these children with chronic conditions.

Most developed countries have community NIV programs that support long-term home NIV use in pediatrics and adults.^6,7^

The previous experiences in different countries, such as France,^8^ the USA,^9^ and Australia,^10^ showed that the initiation of NIV in an outpatient setting in selected patients is safe, feasible, and effective.

Most pediatric centers established outpatient programs to facilitate care and improve adherence. Each program has different clinical pathways and team members, but all agree on providing NIV education and working on mask fitting in the clinics before referring the patients to the sleep laboratory for initiation and titration study.^11,12^

Fauroux et al. described a unique outpatient program of continuous positive airway pressure (CPAP) initiation in selected children with OSA using an integrated team approach.^6^

In the middle east and developing countries, there were no published studies on pediatric NIV outpatient experiences. The shortage of hospital beds availability that was encountered globally during the COVID-19 pandemic had an additional impact on the care of children with chronic diseases.

In our hospital, NIV initiation was exclusively done in the sleep laboratory or, if not available, the pediatric intensive care unit (PICU) for patients’ safety. Due to the lack of hospital bed availability, we had to come up with an alternative setting to prevent delay and provide cost-effective care for these patients. Furthermore, the observed poor sleep efficiency in our complex patients makes successful initiation of NIV during split-night or titration studies very difficult. On January 2020, we started an NIV outpatient program for NIV initiation and patients/parents counseling. The clinic included a pediatric pulmonologist, a nurse coordinator, a respiratory therapist (RT), and a child life specialist. Hence, this study aims to describe the clinical outcome and highlight our experience in implementing the first NIV pediatric outpatient program in the middle east.

## Methods

This study is a retrospective chart review of all patients (below 18 years of age) who were evaluated and treated in the NIV clinic in the pediatric pulmonary clinic at Sidra Medicine - the only national tertiary pediatric center with a specialized pediatric sleep laboratory in Qatar - from January 2020 to November 2021. Patients’ data included demographics, PSG results, and NIV clinic records.

All patients were previously diagnosed with sleep-related breathing disorders (SRBD) which was confirmed by PSG. Patients, therefore, were referred to the NIV clinic by the primary pulmonologist for NIV initiation. Patients were seen in the clinic if they were medically stable and above 1 year of age. The goals of the clinic were to help the patient's family with equipment acquisition, initiate the NIV in an appropriate time interval, educate the caregiver on the NIV application, and troubleshoot issues. The clinic was held once per month during the study period but was closed for four months due to COVID-19 measures that were implemented as a part of Qatar's healthcare plan to limit the spread of COVID-19 which also affected the NIV machine availability.^13^

Upon referral to the NIV clinic, the team reviewed the medical chart and ordered the appropriate NIV equipment and interface based on the patient's age and clinical condition. Afterward, the nurse coordinator would coordinate with the caregivers until the equipment was available, and then the patient would be booked into the clinic. The parents received two reminder calls during the week of the appointment to confirm.

The clinic visits typically lasted three hours and included time for patient monitoring and direct interaction with the patients and their caregivers. During the clinic visit, the care plan and the PSG results were initially explained to the child and/or the caregiver.

The respiratory therapist then helped the caregiver apply the interface without the attached NIV machine. Once the child became comfortable leaving the interface on, the RT would connect the NIV machine and start CPAP mode at a low pressure of 4 _H_2_O_. In children who were planned for CPAP initiation, the pressures were gradually titrated to a maximum of 9 _H_2_O_ as required and tolerated. On the other hand, in children who were planned for Bilevel positive airway pressure (BiPAP) initiation or who required maximum CPAP settings, CPAP was switched to BiPAP mode. The maximum CPAP achieved would be used as the initial positive inspiratory pressure. The PEEP would be kept between 4-6 _H_2_O_. The peak inspiratory pressure (PIP) and positive end-expiratory pressure (PEEP) were increased gradually to a maximum of 16/8 _H_2_O_ as clinically indicated. Finally, an appropriate BiPAP backup rate was set based on the patients’ age. Titration of the settings was monitored using clinical synchrony and the absence of hypoxia. End-tidal carbon dioxide (EtCO_2_) was used in some cases of hypoventilation.

During the interface application and BiPAP titration process, multiple soothing techniques were performed to help patients accept and acclimate to the interface. The techniques included playing favorite video clips and using toys with the help of a child life specialist.

At the end of the clinic, the equipment use and maintenance were re-explained to the caregivers by the RT. Help phone numbers were also provided for troubleshooting and further inquiries.

Follow-up phone calls were performed by the nurse coordinator to check the patient's adherence, parents’ satisfaction, and possible NIV complications. Patients’ adherence was reported by the caregiver (subjective adherence, number of hours NIV was used during the night, and number of nights NIV was used per month). BiPAP sleep titration was requested for all patients.

The need and frequency of follow-up in NIV clinic visits were arranged based on the patient's needs. Otherwise, the patient was referred to the primary pulmonologist.

Finally, patients who had high risks and clinical complexity were identified and referred to pediatric homecare services.

## Results

Twenty-eight patients were seen in our NIV clinic (17 male, 11 female) during the study period. The mean age at NIV initiation was 11 ± 5.17 years. The mean BMI was 32.72 ± 15.91 kg/m^2^. The most common diagnosis was morbid obesity (n = 9, 32.14%), neuromuscular diseases (n = 7, 11%), Prader-Willi syndrome (PWS) (n = 6, 21.42%), trisomy 21 (n = 2, 7.14%), upper airway anomalies (n = 2, 7.14%), and others (n = 2, 7.14%). The sleep-related breathing disorders based on the diagnostic PSG were as follows: obstructive sleep apnea in 21 patients (75%), hypoventilation in 3 patients (11%), mixed apnea in 3 patients (11%), and central apnea in 1 patient (3%), as shown in Table 1.

The median total Apnea-Hypopnea Index (AHI) was 12.7 (Range 0.7-153.9) events per hour. The rest of the sleep-related respiratory parameters are summarized in Table 2.

The median interval between the date of referral to the NIV clinic and the first visit was 32 (Range 1-301) days. The referrals that were delayed were due to either social (family's refusal to initiate the NIV support), or financial (financial difficulties to obtain the medical equipment-NIV machine) issues.

The median number of required NIV visits per patient was 1 (Range 1-3) visits. The reason for the NIV clinic visit was for initiation of NIV in 19/28 patients, to enhance the tolerance and training in 6/28 patients; and for NIV titration in 3/28 patients. The type of initiated respiratory support was BiPAP in 20/28 patients, and 8 patients required CPAP.

The type of interface was nasal in 24/28 patients; Nose-mouth (facial) in 3/28 patients; and nasal prongs in 1/28 patients. The duration of recommended hours of NIV was nocturnal in all patients except for one patient who was on continuous NIV as palliative therapy (BiPAP mode). The median IPAP was 11 (range 6-16) _H_2_O_ and the median EPAP was 6 (range 4-8) _H_2_O_.

During follow-up, six patients were successfully weaned off NIV support by the treating pulmonologist after the resolution of OSA symptoms post adenotonsillectomy and bariatric surgeries. Five patients refused to use NIV at home, 3 of them were adolescents with morbid obesity, 1 patient with PWS and mental retardation, and 1 adolescent patient with down syndrome. Of the remaining 17 patients, 15 patients used NIV ≥  4 hours per night and 13 patients used it ≥  6 hours per night (subjective adherence 88% and 76.5%, respectively). Twelve patients used the NIV for ≥  24 nights per month (70.6%), 4 patients for 12 nights per month (23.5%), and 1 patient used it for 4 nights per month (5.8%). The majority of the patients reported no NIV complications except for 2 patients (nasal redness and eye dryness).

Only nine patients were able to do post-NIV titration PSG due to COVID-19 restrictions.

All parents reported that the clinic experience was beneficial and helped them to be more comfortable in applying the NIV machine to their children at home.

## Discussion

This work describes the first experience of an outpatient NIV initiation program in children with sleep-related breathing disorders in Qatar over 23 months. To date, there has been no published report from the Middle East region. Therefore, it represents pioneering work in this field regionally Before establishing our outpatient NIV program, patients waited up to 2 years for NIV initiation. However, the interval between referral and initiation visit was dramatically decreased to about 1 month in most patients who had no financial or social issues in obtaining the equipment. Literature defines good adherence in the adult population when the equipment is used for at least 4 hours per night for 5 days a week or at least 70% of the days monitored.^14,15^ The definition of Patients’ adherence in the pediatric age group varies in the literature, but most studies used a similar definition to the one applied to adults.^8,16^

In our study, we evaluated patients’ adherence to NIV support depending on parents’ reported feedback due to the COVID-19 restrictions and difficulties in obtaining data recorded by the NIV machines. It is understood that subjective adherence could overestimate patients’ adherence compared to objective adherence which is based on NIV-recorded data download.^17^ Our patients’ reported adherence was excellent. It was estimated to be 88% (as defined as ≥  4 hours per night).

The reported poor adherence in the remaining patients (12%) was attributed to adolescent behavior and mental retardation. Poor adherence to NIV therapy has been reported in children, especially among adolescent and obese patients.^6,17–19^ Difeo et al. prospectively studied the predictors of NIV therapy adherence in children.^20^ In their study, they provided free equipment to exclude any potential economic obstacles. The strongest predictor of poor NIV adherence in their study was lower maternal education. Hawkins et al. reviewed the CPAP-recorded data from 140 patients and determined that 49% were adherent to CPAP.^16^ Only females with developmental delay were associated with better adherence. Factors such as OSA severity and therapeutic pressure did not influence CPAP adherence.

Pediatric outpatient NIV programs have been reported in multiple developed countries and used different approaches and pathways to improve patients’ adherence. Amaddeo et al. showed excellent patient adherence with the use of age and developmental-adjusted interventions and collaboration with trained home care providers.^8^ Previous studies showed better patient adherence when a respiratory therapist (RT) was included in the NIV team.^21^ The CPAP program at The Children's Hospital of Philadelphia (CHOP) showed better patient adherence when shifted to an intensive outpatient CPAP program. The program reported further improvement in adherence after including a behavioral psychologist and respiratory therapist in their program; as well as regular follow-up phone calls.^12^ In our program, the aid of an RT was a cornerstone in the excellent adherence rate and parents’ satisfaction. The RT provided essential education to the patients and their caregivers about equipment and troubleshooting as well as ongoing support/feedback. A child life specialist also helped in the NIV initiation process in our clinic, but in adolescent patients, a behavioral psychologist may aid in this age group.

Our study showed that NIV outpatient programs are achievable and beneficial, especially in developing countries. NIV outpatient programs can help to decrease the load of hospital admissions and sleep laboratories. The programs can also aid in decreasing the waiting time for initiation which can potentially decrease the risk of sleep-related breathing disorder (SRBD) complications.

Home ventilators are currently equipped with built-in software which can provide vital information such as patient's adherence, mask leak, tidal volume, apnea-hypopnea indexes, and percentage of inspirations triggered by the patient. Such data provide objective tools to monitor patients on long-term NIV support.^22^

**Figure 1.**
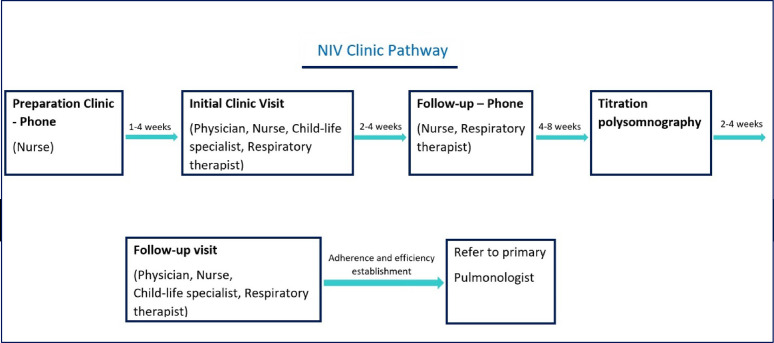
NIV clinic pathway as described in this research.

As a new service, multiple obstacles and identified deficiencies were faced while running the NIV clinics, especially at the time of COVID-19 pandemic and restrictions. The main aim of the clinic was focused on the initiation of NIV support. However, the follow-up visits were limited depending on the patient's needs. Subsequently, a structured referral/follow-up pathway was established to ensure that NIV support was administered safely and efficiently, as shown in Figure 1. 

The Financial burden caused by NIV devices cost was an issue for some patients, which required charity support and social worker referral.

The difficulties in obtaining recorded data from the NIV devices due to COVID-19 restrictions affected our assessment of NIV adherence and efficiency. These data downloads would have decreased the requirement for follow-up titration polysomnography studies.^23^ Therefore, there is still a need to depend on follow-up polysomnography studies to confirm NIV settings and treatment efficiency.

The population of our clinic was mainly represented by children with heterogeneous diseases and ages. These children with complex medical backgrounds would benefit the most from the close monitoring and follow-up by the experienced team in our clinic.

As the only tertiary pediatric center in Qatar, we accept referrals from all hospitals in Qatar. However, future efforts should be made to train other centers in Qatar to start their programs and reach out to more patients and their families. Additionally, studies about cost-effectiveness are needed to evaluate the impact of implementing outpatient NIV programs on sleep laboratories and hospital admission rates.

## Conclusions

Our study reports a favorable outcome of NIV outpatient programs in selected pediatric patients. Despite encountering challenges, it confirms that NIV outpatient programs are achievable and beneficial, especially in developing countries. Our experience also emphasizes the importance of pediatric NIV clinics managed by a specialized team in the initiation and follow-up of NIV support. Additionally, NIV clinics provide alternative pathways for the care of these patients, education, and support of their families, and decrease the load on intensive care and sleep laboratory units.

### Ethical Approvals

The study was approved by the Institutional Review Board of Sidra Medicine (IRB#1839015) on 19/12/2021.

## Figures and Tables

**Table 1.** Demographic Data.

Indicator	Value
Age at the time of visit to NIV clinic (Years), Mean ± SD	11 ± 5.17
Gender, N (%)	N (%)
Male, N (%)	17 (61%)
Female, N (%)	11 (39%)
Body mass index (Kg/m^2^). Mean ± SD	32.72 ± 15.91
**Underlying Disease**	**Value**
Morbid obesity	9 (32.14%)
Neuromuscular diseases	7 (25%)
Prader Willi syndrome	6 (21.42%)
Trisomy 21 Syndrome	2 (7.14%)
Upper airway anomalies	2 (7.14%)
Others Pectus excavatum Methemaglobulinemia	2 (7.14%)

NIV: Non-invasive ventilation; SD: standard deviation

**Table 2.** Baseline polysomnography tests results.

Indicator	Median (Range)
Total Apnea-Hypopnea Index (Events per hour)	12.7 (0.7-153.9)
Type of sleep-related breathing disorder	N (%)
Obstructive sleep apnea Hypoventilation Mixed sleep apnea Central sleep apnea	21 (75%) 3 (11%) 3 (11%) 1 (3%)
**Indicator**	**Median (Range)**
Average oxygen saturation (%)	95 (90-99)
Lowest oxygen saturation (%)	80 (33-92)
Time spent with oxygen saturation < 90% (%)	0.99 (0-47.64)
Oxygen Desaturation Index (Events per hour)	20.45 (2.87-156.81)
Mean End-tidal PCO_2_ (EtCO_2_) (mmHg)	42.5 (32-52)
Highest End-tidal PCO_2_ (mmHg)	48 (42-54)
Time spent with EtCO_2_ > 50 mmHg (%)	0 (0-3.4)

PCO_2_: Partial pressure of carbon dioxide

**Table 3.** NIV Clinic Data.

Indicator	Median (Range)
Number of required visits per patient (Visits)	1 (1-3)
**Indication of NIV clinic referral**	**N (%)**
Initiation	19 (68%)
Enhance tolerance to NIV	6 (21%)
Titration of NIV settings	3 (11%)
**Type of established respiratory support**	
BiPAP	20 (71%)
CPAP	8 (29%)
**The type of NIV interface**	
Nasal	24 (85.7%)
Nose-Mouth (Facial)	3 (10.7%)
Nasal Prongs	1 (3.6%)
**Duration of recommended NIV support at home**	
Nocturnal	27 (97%)
Continuous	1 (3%)
**Indicator**	**Median (Range)**
Inspiratory positive airway pressure (IPAP) cmH2O	11 (6-16)
Expiratory positive airway pressure (IPAP) cmH2O	6 (4-8)

NIV: Non-invasive ventilation; BiPAP: Bilevel positive airway pressure; CPAP: Continuous positive airway pressure.
